# Calcium ions in tap water may increase the adhesion ability of *Acanthamoeba*, potentially enhancing its cytopathic effects on corneal cells

**DOI:** 10.1051/parasite/2025066

**Published:** 2025-11-24

**Authors:** Yu-Jen Wang, Yao-Tsung Chang, Tsun-Hsien Hsiao, Chun-Hsien Chen, Chih-Ming Tsai, Jian-Ming Huang

**Affiliations:** 1 Department of Parasitology, School of Medicine, China Medical University Taichung 406040 Taiwan; 2 Department of Biochemistry and Molecular Biology, College of Medicine, National Cheng Kung University Tainan 701 Taiwan; 3 School of Medicine, College of Life Sciences and Medicine, National Tsing Hua University Hsinchu 300044 Taiwan; 4 Department of Medical Science, College of Life Sciences and Medicine, National Tsing Hua University Hsinchu 300044 Taiwan; 5 Institute of Molecular and Cellular Biology, College of Life Sciences and Medicine, National Tsing Hua University Hsinchu 300044 Taiwan; 6 Department of Parasitology, College of Medicine, Chang Gung University Taoyuan 333323 Taiwan; 7 Department of Parasitology, College of Medicine, National Cheng Kung University Tainan 701 Taiwan; 8 Department of Physiology, College of Medicine, National Cheng Kung University Tainan 701 Taiwan

**Keywords:** *Acanthamoeba*, *Acanthamoeba* keratitis, Domestic tap water, Adhesion

## Abstract

*Acanthamoeba* spp. are free-living amoebae found in various water sources, including domestic tap water. These amoebae are known to cause *Acanthamoeba* keratitis, a severe corneal infection that can lead to vision loss. Although *Acanthamoeba* keratitis is commonly associated with water exposure, the environmental factors that enhance *Acanthamoeba* adhesion to corneal epithelial cells remain poorly understood. In this study, we examined the effects of domestic tap water on *Acanthamoeba* adhesion and found that exposure to tap water significantly increased the number of adherent trophozoites, potentially enhancing their cytopathic effects on corneal cells. We hypothesized that the calcium ions present in tap water may play a key role in regulating amoebal adhesion. To investigate this further, we analyzed the expression levels of several adhesion-related genes following exposure to different water conditions. Our findings suggest that tap water facilitates *Acanthamoeba* attachment and may contribute to disease progression. Understanding the role of calcium and other potential regulatory factors in adhesion may provide new insights into *Acanthamoeba* keratitis pathogenesis and aid in the development of preventive strategies.

## Abstract (fr)

Les *Acanthamoeba* spp. sont des amibes libres présentes dans diverses sources d’eau, notamment l’eau du robinet. Ces amibes sont connues pour provoquer la kératite à *Acanthamoeba*, une infection cornéenne grave pouvant entraîner une perte de vision. Bien que la kératite à *Acanthamoeba* soit fréquemment associée à l’exposition à l’eau, les facteurs environnementaux qui favorisent l’adhérence d’*Acanthamoeba* aux cellules épithéliales cornéennes restent mal compris. Dans cette étude, nous avons examiné les effets de l’eau du robinet sur l’adhérence d’*Acanthamoeba* et constaté que l’exposition à cette eau augmentait significativement le nombre de trophozoïtes adhérents, renforçant potentiellement leurs effets cytopathiques sur les cellules cornéennes. Nous avons émis l’hypothèse que les ions calcium présents dans l’eau du robinet pourraient jouer un rôle clé dans la régulation de l’adhérence des amibes. Afin d’approfondir cette hypothèse, nous avons analysé les niveaux d’expression de plusieurs gènes liés à l’adhérence après exposition à différentes conditions d’eau. Nos résultats suggèrent que l’eau du robinet favorise l’adhérence d’*Acanthamoeba* et pourrait contribuer à la progression de la maladie. Comprendre le rôle du calcium et d’autres facteurs de régulation potentiels dans l’adhérence pourrait apporter de nouvelles perspectives sur la pathogenèse de la kératite à *Acanthamoeba* et contribuer à l’élaboration de stratégies préventives.

## Introduction

*Acanthamoeba* sp. is a pathogenic protozoan that exists as a free-living amoeba (FLA) in diverse environments, including lakes, swimming pools, soil, and airborne dust [5, 10]. *Acanthamoeba* are classified into 23 genotypes, ranging from T1 to T23, based on subgenus classification and taxonomy. This classification is determined by the nucleotide sequence of 18S ribosomal RNA, with a sequence divergence of at least 5% between different genotypes [36, 39]. Among these, several genotypes, including T2, T3, T4, T5, T6, T11, and T15, have been identified as pathogenic, with T4 being the most commonly isolated genotype from both human and environmental samples [28]. Homologous analysis of 18S rRNA from 34 *Acanthamoeba* isolates collected from rivers and drinking water in southern Taiwan revealed the presence of seven genotypes: T4, T5, T6, T7/T8, T11, T12, and T15. Notably, all these genotypes, except T7/T8 and T12, have been linked to *Acanthamoeba*-related diseases [20]. The widespread presence of *Acanthamoeba* in such environments poses a continuous risk of infection, particularly among contact lens (CL) users.

*Acanthamoeba* is responsible for severe infections such as granulomatous amoebic encephalitis and *Acanthamoeba* keratitis (AK) [1, 3, 5, 6, 29, 41]. Infected patients may experience symptoms such as lid edema, photophobia, epithelial defects, and characteristic ring-shaped stromal infiltrates, often triggered by corneal injuries [24, 26]. *Acanthamoeba* colonization primarily occurs in the homes of patients with infectious keratitis, as evidenced by the frequent isolation of the organism from domestic tap water. In Busan, Korea, FLA were detected in 47% (97/207) of domestic tap water samples, with *Acanthamoeba* specifically identified in 6% (16/207) of these samples [16]. Additionally, *Acanthamoeba* has been reported as a common contaminant in CL storage cases in the Philippines [37]. This indicates that domestic tap water is a significant reservoir for this microorganism and a potential common source of infection.

The adhesion of *Acanthamoeba* to CL surfaces is a critical initial step in AK pathogenesis. Studies have shown that trophozoites adhere to CL materials at a significantly higher rate than cysts [21, 23]. Remarkably, even brief exposure to a minimally contaminated solution or environment is sufficient for trophozoites to rapidly attach to CL [12, 18]. The infection process begins with the interaction between the mannose-binding protein (MBP) on the surface of *Acanthamoeba* and mannosylated proteins present on host cells [11]. In addition, *Acanthamoeba* can adhere to laminin in the Bowman’s membrane and the extracellular matrix through its laminin-binding protein (AhLBP), a crucial factor that facilitates invasion of the corneal stroma during AK [13]. Following adhesion, *Acanthamoeba* secretes a range of proteolytic enzymes that degrade host tissues, leading to inflammation, tissue edema, and necrosis [33]. Among these enzymes, serine proteases and cysteine proteases play a predominant role in tissue hydrolysis and pathogenesis [22, 32].

The treatment of AK remains a considerable challenge. Although trophozoites are generally susceptible to antibiotics, cysts exhibit remarkable resistance [14]. Polyhexamethylene biguanide (PHMB), a polymeric biocide, is widely used as a disinfectant and antiseptic agent for AK treatment. Recent studies suggest that PHMB 0.08% monotherapy may be as effective as a combination of PHMB 0.02% with propamidine [8]. PHMB exhibits antimicrobial activity against a broad range of pathogens, including *Pseudomonas aeruginosa*, *Staphylococcus aureus*, *Escherichia coli*, *Candida albicans*, and *Aspergillus brasiliensis* [2, 19, 30, 43, 46]. Its mechanism of action involves the binding of highly charged positive molecules to the negatively charged phospholipid bilayer of microbial cell membranes, leading to membrane penetration, structural damage, cell lysis, and ultimately pathogen death [25]. The combination of PHMB with propamidine and neomycin has been shown to improve AK symptoms in all treated patients, with pain reduction observed within two to four weeks [40]. Moreover, PHMB is incorporated into CL cleaning solutions, often in combination with hydrogen peroxide, to prevent corneal infections [34]. In cases where topical treatments fail, corneal transplantation may be considered; however, this approach carries the risk of incomplete removal of all trophozoites or cysts from the transplanted cornea [9]. Consequently, no standardized therapeutic strategy has yet been established for routine clinical practice.

In this study, we explored how the adhesion ability of *Acanthamoeba* may be enhanced upon its exposure to domestic tap water, facilitating its attachment to CLs or corneal surfaces. Furthermore, we suggest that *Acanthamoeba* may utilize proteases to degrade the extracellular matrix (ECM) of corneal cells after adhesion, ultimately increasing its ability to establish an infection. Understanding these mechanisms provides valuable insights into the pathogenicity of *Acanthamoeba* and highlights the potential risks associated with contaminated water sources, emphasizing the need for improved disinfection strategies for CL care.

## Materials and methods

### *Acanthamoeba castellanii* cultivation

Trophozoites of *A. castellanii* (Neff strain, ATCC No. 30010, Pacific Grove, CA, USA) were axenically cultured at 28 °C in peptone-yeast extract-glucose (PYG) medium (20 g/L proteose peptone, 2 g/L yeast extract, 0.1 M glucose, 4 mM MgSO_4_, 3.4 mM sodium citrate, 0.9 mM Fe(NH_4_)_2_(SO_4_)_2_, 1.3 mM Na_2_HPO_4_, and 2 mM K_2_HPO_4_, pH 6.5) in cell culture flasks [15].

### Amoeba adhesion assay

*Acanthamoeba* adhesion was assessed by incubating 2 × 10^6^/mL trophozoites under different treatment conditions, including PYG medium, Page’s Amoeba Saline (PAS) (containing 120 mg NaCl, 4 mg MgSO_4_ × 7H_2_O, 3 mg mM CaCl_2_, 142 mg Na_2_HPO_4_, and 136 mg KH_2_PO_4_, domestic water, 50 mM calcium chloride (CaCl_2_), or double-deionized water (DDW) for 1 h at 28 °C. Following incubation, non-adherent cells were removed by washing three times with PAS. The adhesion percentage was quantified by counting the number of attached *Acanthamoeba* cells under a phase-contrast microscope.

### Cell culture

Statens Serum Institut Rabbit Cornea (SIRC) cells were purchased from the Bioresource Collection and Research Center and cultured in Dulbecco’s modified Eagle’s medium (DMEM) supplemented with 10% heat-inactivated fetal bovine serum and 1% penicillin-streptomycin. The cells were maintained as monolayers at 37 °C with 5% CO_2_ in an incubator. To prepare for cytopathic assays, cells were cultured overnight to form monolayers, and the following day the culture medium was replaced with serum- or antibiotic-free DMEM.

### Cytopathic effect

SIRC cells were seeded into 24-well plates and maintained as confluent monolayers. Before infection, the culture medium was replaced with fresh DMEM. For the infection assay, SIRC monolayers were incubated with an equal volume of serum-free DMEM (control) or *Acanthamoeba* trophozoites (2 × 10^5^ cells/well) that had been pretreated under different conditions, such as PYG medium, PAS, domestic tap water, 50 mM CaCl_2_, or DDW for 1 h at 37 °C with 5% CO_2_. After incubation, non-adherent *Acanthamoeba* cells were removed by washing the wells three times with phosphate-buffered saline (PBS). The SIRC monolayers with adherent amoebae were then maintained in serum-free DMEM for an additional 24 h at 37 °C with 5% CO_2_. Following the 24-hour infection period, the cell layers were fixed with 2% paraformaldehyde. Cell imaging was performed using a ZOE Fluorescent Cell Imager (Bio-Rad, Hercules, CA, USA).

### Total RNA isolation and cDNA synthesis

A Direct-zol RNA kit (Cat. No. R2052, Zymo Research, Irvine, CA, USA) was used to extract RNA. The concentration and A260/A280 ratio of the mRNA were determined using a NanoDrop One (Thermo Fisher Scientific, Waltham, MA, USA). A Moloney Murine Leukemia Virus (MMLV) reverse transcription kit (Protech Technology Enterprise, Taipei, Taiwan) was used for cDNA synthesis. Reverse transcription was conducted in a 20 μL reaction volume, under the following conditions: 25 °C for 10 min, 42 °C for 60 min, and 70 °C for 10 min.

### Polymerase chain reaction (PCR)

The *Acanthamoeba* actin related protein 2 (ARP2) forward primer sequence was 5′–GCT GTC TTG ACC CTC TAC GC–3′, paired with a reverse primer sequence of 5′–AGC GAG AAG CCC TCG TAC AC–3′, resulting in a 101 bp PCR product [33]. The MBP forward primer sequence was 5′–AGG GCG AGA CCT ACG ATA GC–3′, with a reverse primer sequence of 5′–CCT CGT AGA CGA AGG TGA GG–3′, yielding a 165 bp amplification product [33]. The forward primer sequence for the AhLBP was 5′–CCA ACA CCG ACT CTC CTC TC–3′, paired with a reverse primer sequence of 5′–CTC CTC AGG GTC ACG GTA GA–3′, generating a 183 bp amplification product [33]. For cysteine protease 3 (CP3), the forward primer sequence was 5′–CGA TGC CTC GCA CAA CTC CTT C–3′, and the reverse primer sequence was 5′–CCA CGA GTT CTT GAC GAG CCA–3′, resulting in a 148 bp PCR product [45]. The serine endopeptidase (SEP) forward primer sequence was 5′–GAA GGC GCT CAC CGA ATA CAT–3′, paired with a reverse primer sequence of 5′–GGT TCG TCA TCT GCT GAT AGC C–3′, resulting in a 104 bp amplification product [31]. The PCR products were separated on a ClearVision DNA Stain (Protech) gel using agarose gel electrophoresis. All experiments were performed independently in triplicate. Image analysis and quantification were performed using Bio-1000F (Microtek, International, Inc., Hsinchu, Taiwan) and ImageJ software, respectively.

### Statistical analysis

Data are presented as the mean ± standard deviation (SD) from three independent experiments. Statistical analysis was performed using the Student’s *t*-test, with a *p*-value < 0.05 deemed statistically significant.

## Results

### Adhesion percentage of *Acanthamoeba* after treatment with different water conditions

**Figure 1:**
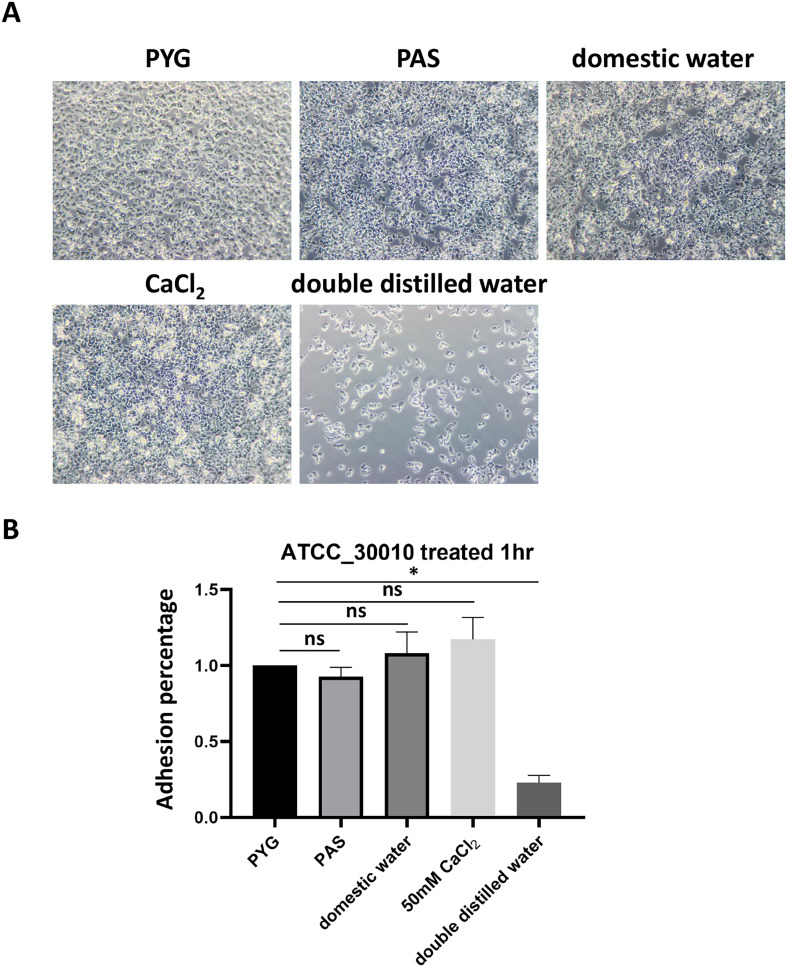
Adhesion percentage of *Acanthamoeba* after treatment with different water conditions. (A) *Acanthamoeba* was incubated with PYG medium, PAS, domestic tap water, 50 mM CaCl_2_, or DDW for one hour and observed through microscopy. (B) The adhesion percentage of *Acanthamoeba* incubated with PYG medium, PAS, domestic tap water, CaCl_2_ and DDW were examined. Bar graphs depict the mean ± SD (*p ≤ 0.05).

*Acanthamoeba* colonization primarily occurs in the homes of patients with infectious keratitis, as evidenced by the frequent isolation of the organism from domestic tap water. To determine whether *Acanthamoeba* exhibits enhanced adhesion in domestic tap water, we compared its adhesion ability in plates after incubation in PYG medium, PAS, and domestic tap water. The results showed no significant differences in the adhesion percentage of *Acanthamoeba* treated with domestic tap water, PYG medium, or PAS (Figs. 1A and 1B). Because domestic tap water contains various ions, including calcium chloride, we further investigated the effect of ions on *Acanthamoeba* adhesion using 50 mM CaCl_2_ and DDW. The results revealed that *Acanthamoeba* treated with DDW exhibited a significantly reduced adhesion percentage; approximately 20% of that seen following treatment with PYG medium, PAS, and domestic tap water. However, when CaCl_2_ was added to DDW, the adhesion percentage increased to over 100%, showing no significant difference from the adhesion percentage observed in PYG medium, PAS, and domestic tap water (Figs. 1A and 1B). These findings suggest that the ions present in domestic tap water may influence *Acanthamoeba* adhesion, potentially increasing the risk of infection.

### Adhesion of *Acanthamoeba* to SIRC cells after pretreatment in different water conditions

**Figure 2:**
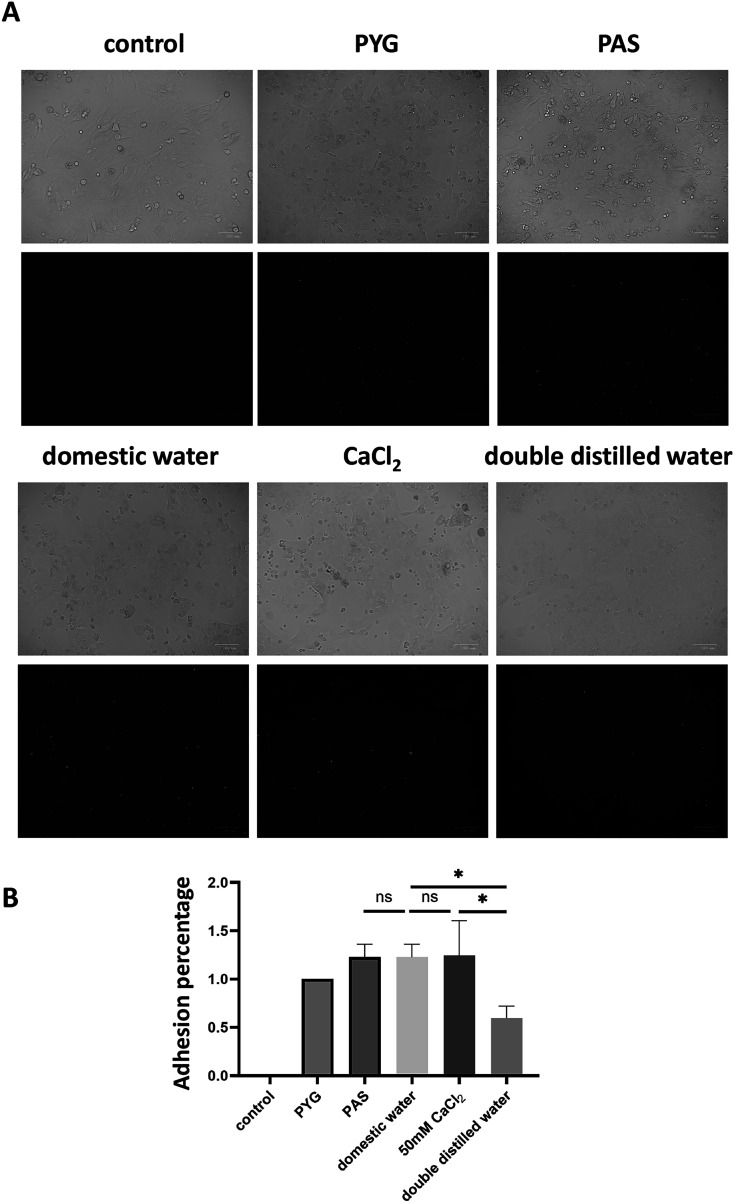
Adhesion of *Acanthamoeba* to SIRC cells after pretreatment with different water conditions. (A) *Acanthamoeba* trophozoites were incubated with PYG medium, PAS, domestic tap water, CaCl_2_, or DDW, followed by staining with calcein AM (green) for one hour. The stained amoebae were then co-cultured with SIRC cells. After incubation, fluorescence microscopy was performed to assess adhesion. The upper panel shows bright-field images, while the lower panel displays dark-field fluorescence images, where green fluorescence indicates *Acanthamoeba*. (B) The adhesion percentage of *Acanthamoeba* were examined after pretreatment with PYG medium, PAS, domestic tap water, CaCl_2_, or DDW subsequent incubation with SIRC cells. Bar graphs depict the mean ± SD (*p ≤ 0.05).

**Figure 3:**
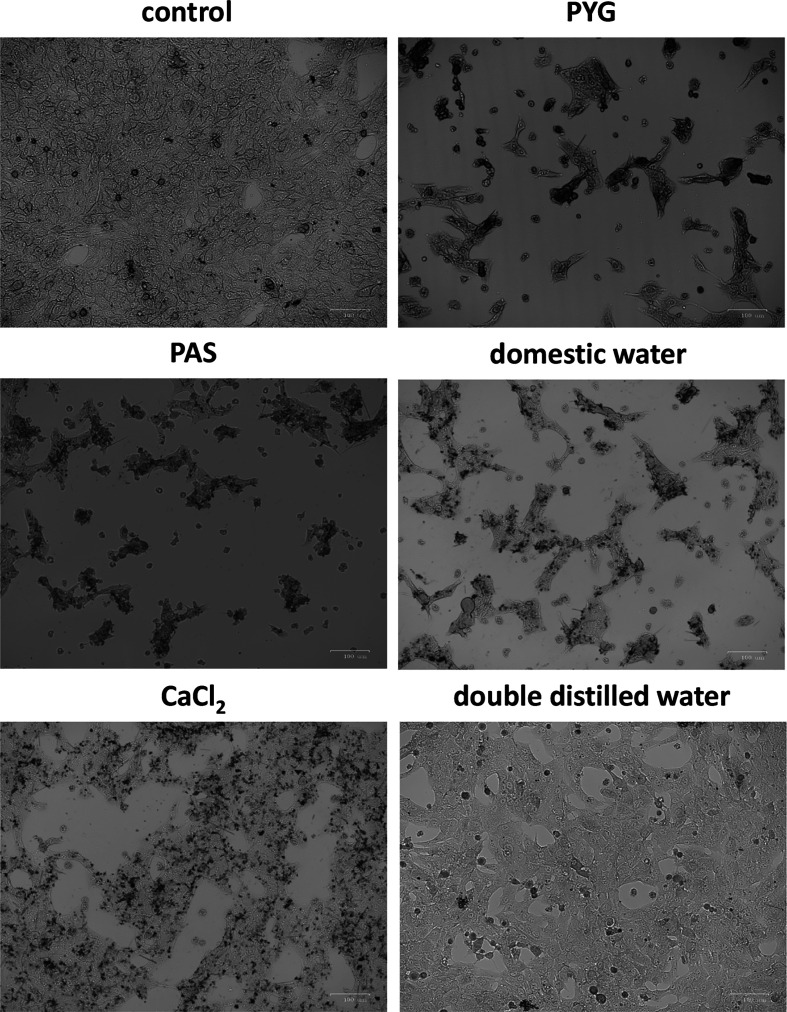
The cytopathic effect on SIRC cells of *Acanthamoeba* pretreated with different water conditions. SIRC monolayers with adherent *Acanthamoeba* pretreated with PYG medium, PAS, domestic tap water, CaCl_2_, or DDW were incubated for 24 h. Following the 24 hour-infection period, the cell layers were analyzed using a ZOE Fluorescent Cell Imager (Bio-Rad).

To investigate whether *Acanthamoeba* treated with domestic water exhibits enhanced adhesion to corneal cells, potentially increasing the risk of AK, we pretreated *Acanthamoeba* with different water conditions, followed by calcein AM staining, and incubation with SIRC cells for one hour. After incubation, non-adherent amoebae were removed by washing. Fluorescence microscopy revealed that *Acanthamoeba* pretreated with domestic water exhibited greater adhesion to SIRC cells than those pretreated with DDW. Notably, when CaCl_2_ was added to DDW, *Acanthamoeba* adhesion increased compared with DDW alone (Fig. 2A). Quantitative analysis showed no significant differences in adhesion percentage among the domestic tap water-, PAS-, and CaCl_2_-treated groups compared to the PYG medium group. However, the adhesion percentage of the DDW-treated group was significantly lower than those of the domestic tap water- and CaCl_2_- treated groups (Fig. 2B). To further determine whether the *Acanthamoeba* adhesion percentage affects corneal cell damage, amoebae pretreated with different water conditions were incubated with SIRC cells for one hour, followed by the removal of non-adherent cells. The cultures were then incubated for 24 h to assess cytopathic effects. The results indicated that SIRC cells in the domestic water, PAS, CaCl_2_, and PYG treatment groups exhibited significant cellular damage compared to the control group. In contrast, the DDW-treated group showed no significant damage to SIRC cells compared to the control group (Fig. 3). These findings suggest that *Acanthamoeba* present in domestic water exhibits enhanced adhesion to corneal cells, facilitating its proliferation after attachment, and potentially increasing the likelihood of AK.

### Expression of virulence factors after *Acanthamoeba* treatment with different water conditions

**Figure 4:**
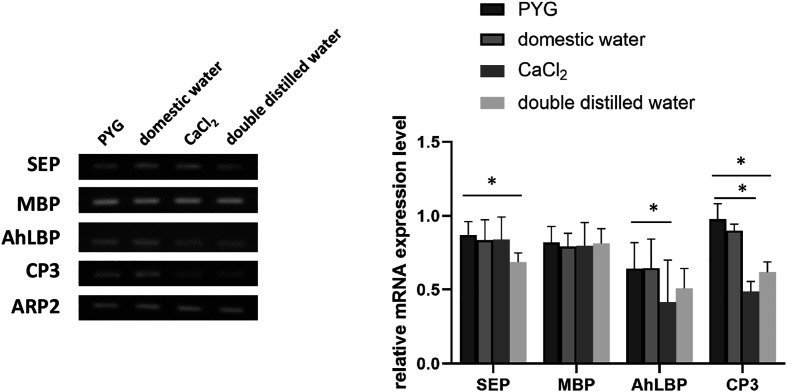
Expression of virulence factors in *Acanthamoeba* pretreated with different water conditions. The relative mRNA expression levels of *SEP*, *MBP*, *AhLBP*, and *CP3* in *Acanthamoeba* pretreated with PYG medium, PAS, domestic tap water, CaCl_2_, or DDW for one hour were quantified using ImageJ software. The expression levels of the *ARP2* gene served as an internal control. Bar graphs represent the mean ± SD (*p* ≤ 0.05).

To investigate whether *Acanthamoeba* exposure to tap water alters its virulence factors, thereby enhancing its adhesion to and destruction of corneal cells, we examined the expression of two adhesion-related proteins (MBP and AhLBP) and two proteases (SEP and CP3) (Fig. 4). This revealed that MBP expression did not differ significantly between groups. For AhLBP, there was no significant difference between the PYG and tap water-treated groups, whereas its expression was significantly lower in the CaCl_2_ groups than in the PYG group. Regarding protease expression, SEP showed no significant differences among the PYG, tap water, and CaCl_2_ groups, but its expression was significantly lower in the DDW group than in the PYG group. In contrast, CP3 expression was not significantly different between the PYG and tap water-treated groups, but was significantly decreased in the CaCl_2_ and DDW groups compared to the PYG group. These findings suggest that *Acanthamoeba* can maintain its adhesion and virulence capabilities in tap water, meaning that contaminated water supplies can harbor *Acanthamoeba* capable of infecting corneal cells.

## Discussion

### The role of ions in enhancing *Acanthamoeba* adhesion

The adhesive ability of *Acanthamoeba* is a critical factor in the development of infectious keratitis. Our study demonstrated that domestic tap water did not significantly enhance *Acanthamoeba* adhesion compared to PYG medium and PAS, suggesting that the basic adhesion properties of the amoebae remain stable under these conditions. However, we observed a significant reduction in adhesion when *Acanthamoeba* were treated with DDW, which lacks ions. Interestingly, the addition of CaCl_2_ to DDW restored the adhesion capacity of *Acanthamoeba* to levels comparable to that in domestic tap water, PYG medium, and PAS. These results indicate that cations may play a crucial role in facilitating *Acanthamoeba* adhesion to surfaces. In protozoa, calcium regulates various cellular processes and plays a crucial role in the signaling pathways that drive growth and development [38]. Calcium ions are known to mediate cell adhesion by interacting with surface proteins and extracellular matrix components. Previous studies have demonstrated that calcium ions enhance *Acanthamoeba* adhesion to extracellular matrix proteins in a dose-dependent manner [44]. It has also been reported that calcium ions significantly influence *Acanthamoeba* adhesion and motility, as 1 mM or 0.5 mM CaCl_2_ maintained adhesion and locomotion, whereas reducing the concentration to 0.1 mM caused detachment and loss of movement [35]. It is plausible that a similar mechanism enhances *Acanthamoeba* adhesion in the presence of tap water containing ions. This finding suggests that the presence of these ions in domestic water could contribute to increased infection risk by promoting amoeba adhesion to corneal surfaces and CLs.

### Influence of water treatment on *Acanthamoeba* adhesion to corneal cells and cytopathic effects

Previous studies have indicated that water storage tanks facilitate the colonization of FLA, including *Acanthamoeba*, in domestic tap water. [17]. The pathogenesis of AK begins with the adherence of trophozoites to the corneal epithelium. Given the importance of adhesion in *Acanthamoeba* pathogenicity, we examined whether amoebae pretreated under different water conditions exhibited differential adhesion to SIRC cells. Our findings demonstrate that amoebae pretreated with domestic water exhibit significantly greater adhesion to SIRC cells than those pretreated with DDW. Consistent with our previous results, calcium chloride supplementation restored adhesion in DDW-treated amoebae (Figs. 2A and 2B). A previous study showed that geographical differences in the incidence of AK in the United Kingdom may be influenced by water hardness. Individuals receiving hard water had a three-fold higher risk of developing AK than those receiving soft water.[42]. These results highlight the role of environmental ions in facilitating *Acanthamoeba* attachment to corneal surfaces. We also assessed the cytopathic effects of *Acanthamoeba* adhesion on SIRC cells. Our results revealed that amoebae treated with domestic water, PAS, CaCl_2_, and PYG medium induced significant cellular damage after 24 h of co-incubation. In contrast, DDW-treated *Acanthamoeba* caused minimal damage, indicating reduced pathogenic potential in the absence of ions (Figs. 3A and 3B). The ability of *Acanthamoeba* to adhere to corneal cells is a prerequisite for cytopathogenicity, because adhesion facilitates nutrient acquisition and cytotoxic enzyme secretion [27]. The observed reduction in adhesion and cytopathic effects in DDW-treated amoebae suggests that ion depletion diminishes amoeba virulence, possibly by affecting adhesion protein expression and enzymatic activity [35, 44]. These findings reinforce the hypothesis that ions in domestic tap water contribute to the pathogenic potential of *Acanthamoeba* and may play a role in the transmission of infection.

### Virulence factor expression in *Acanthamoeba* under different water conditions

To further elucidate the mechanisms underlying the observed adhesive and cytopathic effects, we examined the expression of adhesion-related proteins (MBP and AhLBP) and proteases (SEP and CP3). The primary *Acanthamoeba* adhesin is MBP, a lectin-like glycoprotein found on the trophozoite surface that binds to mannose residues on host cell glycoproteins [7]. MBP expression remained unchanged across all treatment groups, suggesting that this protein was not significantly influenced by water composition. AhLBP is a non-integrin laminin receptor and was the first LBP gene to be cloned and characterized from parasitic protozoans [13]. AhLBP expression was significantly reduced in the CaCl_2_ groups compared to the PYG group, indicating that this adhesion protein is not modulated by ion availability. This finding suggests that AhLBP may contribute to the enhanced adhesion observed in tap water-treated *Acanthamoeba* due to other environmental factors such as chlorine [4]. Regarding protease activity, SEP expression remained stable across the PYG, tap water, and CaCl_2_ groups, but was significantly reduced in the DDW group. However, CP3 expression showed no difference between the PYG and tap water groups but was significantly decreased in the CaCl_2_ and DDW groups. Previous studies have shown that exposure to sublethal concentrations of chlorine increases the cytotoxicity of *Acanthamoeba* and upregulates the expression of its virulence factor, CP3 [4]. Virulence factor CP3 is unlikely to contribute to the increased virulence of *Acanthamoeba* through ion-mediated mechanisms. Instead, its upregulation may have been induced by the chlorine present in the tap water. These results suggest that while *Acanthamoeba* maintains the expression of virulence factors such as SEP and MBP in tap water, its proteolytic activity is diminished in the absence of ions. Because proteases are essential for degrading host tissue and facilitating invasion [27], the observed reduction in SEP and CP3 expression in DDW-treated amoebae could contribute to their decreased cytopathogenicity (Fig. 3). This further supports the hypothesis that ions and chlorine enhance *Acanthamoeba* virulence by maintaining protease activity. Overall, our findings highlight the role of environmental factors in modulating the virulence and adhesion of *Acanthamoeba*. The presence of ions in tap water appears to enhance amoebal adhesion to corneal cells, promote protease activity, and maintain virulence factor expression, thereby increasing the risk of AK. These results emphasize the need for improved water hygiene practices, particularly for individuals at risk of infection such as CL wearers. Future studies should investigate the specific molecular mechanisms by which ions regulate *Acanthamoeba* adhesion and virulence, as well as explore potential strategies to mitigate waterborne transmission risks.

## Conclusion

Tap water enhances *Acanthamoeba* adhesion to corneal cells, potentially increasing their cytopathic effects and contributing to AK pathogenesis. Calcium ions may be a key factor in this process. These findings highlight the need for greater awareness of environmental risk factors and may inform future strategies for AK prevention and intervention.

## Data Availability

Data supporting the conclusions of this article are included within the article. The datasets used and/or analyzed during the present study are available from the corresponding author upon reasonable request.
